# Assessment of 5-Aminolevulinic Acid-Mediated Photodynamic Therapy on Bone Metastases: An in Vitro Study

**DOI:** 10.3390/biology10101020

**Published:** 2021-10-09

**Authors:** Saskia Magdalen Sachsenmaier, Frank Traub, Anna Cykowska, Rosa Riester, Nikolaus Wülker, Christian Walter, Marina Danalache

**Affiliations:** 1 Laboratory of Cell Biology, Department of Orthopedic Surgery, University Hospital of Tübingen, 72072 Tübingen, Germany; Anna.cykowska@edu.unito.it (A.C.); rosa.riester@med.uni-tuebingen.de (R.R.); niko.wuelker@med.uni-tuebingen.de (N.W.); marina.danalache@med.uni-tuebingen.de (M.D.); 2 Department of Orthopedic Surgery, University Hospital of Tübingen, 72072 Tübingen, Germany; christian.walter@med.uni-tuebingen.de; 3 Department of Orthopedics and Traumatology, University Medical Center Mainz, Johannes Gutenberg University Mainz, 55131 Mainz, Germany; frank.traub@unimedizin-mainz.de; 4 Department of Clinical and Biological Sciences, University of Turin, 10043 Orbassano, Italy

**Keywords:** bone, metastasis, photodynamic therapy, photosensitizer, migration, viability, apoptosis, senescence, minimally invasive

## Abstract

**Simple Summary:**

Bone metastases are typically associated with a short-term prognosis. Photodynamic therapy (PDT) emerges as a promising alternative treatment for targeting metastatic lesions. In this study we investigated the effect of 5-aminolevulinic acid-mediated PDT treatment on both primary and human bone metastatic cancer cell lines. We found that human cell lines have different sensitivity to the same doses and exposure of 5-ALA PDT resulting in two different cell fates, apoptosis or senescence, depending on the extent of the cellular damage. As such, PDT has potential applicability in bone metastases of invasive ductal carcinoma.

**Abstract:**

Bone is a frequent site of metastases, being typically associated with a short-term prognosis in affected patients. Photodynamic therapy (PDT) emerges as a promising alternative treatment for controlling malignant disease that can directly target interstitial metastatic lesions. The aim of this study was to assess the effect induced by PDT treatment on both primary (giant cell bone tumor) and human bone metastatic cancer cell lines (derived from a primary invasive ductal breast carcinoma and renal carcinoma). After 24 h post light delivery (blue light-wavelength 436 nm) with 5-aminolevulinic acid, the effect on cellular migration, viability, apoptosis, and senescence were assessed. Our results showed that bone metastasis derived from breast cancer reacted with an inhibition of cell migration coupled with reduced viability and signs of apoptosis such as nuclei fragmentation following PDT exposure. A limited effect in terms of cellular viability inhibition was observed for the cells of giant cell bone tumors. In contrast, bone metastasis derived from renal carcinoma followed a different fate—cells were characterized by senescent features, without a notable effect on cell migration or viability. Collectively, our study illustrates that PDT could act as a successful therapy concept for local tumor control in some entities of bone metastases.

## 1. Introduction

The incidence of bone metastases in patients with advanced tumor disease has increased in recent decades. After the lung and liver, bone is the third most affected organ by tumor metastases [1]. The clinical landscape of bone metastases is challenging as they cause severe pain, bone fractures, spinal cord compression, and implicitly, a sustained degradation of the quality of life [2]. Currently, radiotherapy is the gold standard for the treatment of bone metastasis. However, it offers limited and temporary palliation of pain and can adversely affect the capacity for soft tissue to repair post-treatment. Additionally, it has been estimated that peripheral nerve damage that leads to progressive muscle denervation and atrophy occurs in 7–13% of patients after radiation therapy [3,4].

Photodynamic therapy (PDT) has been emerging as a promising way to treat and control malignant diseases [5,6,7]. The PDT basic setup is comprised of three essential components: photosensitizer (which is preferentially taken up by tumor cells), light source, and oxygen [8]. Even though none of the individual components are toxic, their combination initiates a photochemical reaction culminating with the generation of singlet oxygen that produces a local cytotoxic effect [9]. An asset of PDT treatment is the possibility to place the optical fiber cable of the light source direct or immediately adjacent to the lesion. This approach not only enables targeted tumor destruction but also spares the surrounding soft tissue from collateral damage without interfering with the subsequent wound healing processes [10]. PDT can be employed either complementary to radiotherapy, which is better suited for treating larger tumors [11], or a stand-alone therapy. Different kinds of PDT approaches are currently available, differing in terms of light source or photosensitizer. The latter directly influences the efficiency of therapy based on its delivery mode and targeting ability. In cancer therapy, the majority of employed photosensitizers have a tetrapyrrole structure, similar to that of the protoporphyrin contained in hemoglobin. A new chapter for PDT was sketched in 1987 by Malik and Lugaci discovery that 5-aminolevulinic acid (5-ALA) is a biosynthetic precursor of the photosensitizer protoporphyrin IX [12]. 5-ALA is considered a “pro-drug” and requires to be metabolically converted to protoporphyrin in order to become an active photosensitizer [13]. Thus, 5-ALA can be topically applied to tissues, or even administered orally, significantly reducing adverse phototoxic effects and without the risk of degradation [14]. After oral administration 5-ALA is absorbed relatively rapidly from the gastrointestinal tract and taken up and metabolized within the cytosol, driving the enzymatic reactions to increase protoporphyrin IX production [15]. Moreover, 5-ALA is a Food and Drug Administration (FDA)- and European Medicine Agency (EMA)-approved photosensitizer and licensed for in vivo applications.

Despite the fact that PDT is used in brain [16,17], lung [18], and prostate cancer [19], it is mainly indicated for early carcinomas without detectable metastases [20] due to low drug delivery efficiency. In the context of vertebral tumors, PDT was not only effective in ablating cancerous lesions [21], but also in enhancing the vertebral structure (particularly when combined with bisphosphonates) [22]. In fact, a recently completed phase I clinical trial showed that the combination of vertebral metastasis PDT and vertebral cement augmentation is not only safe from a pharmaceutical and neurologic perspective but also proved highly effective in pain reduction [23]. Such promising results further advocate for a scale-up study to evaluate the potential PDT efficacy in metastatic treatment [23]. Until recently, the efficacy of PDT in treating a deep-seated tumor was limited due to a low light penetration depth, low oxygen concentration in the hypoxic core, and poor photosensitizer accumulation inside the tumor. The potential solution is the advancement of nanotechnology, that is, nanoparticles that carry photosensitizers as well as new techniques and strategies such as two-photon excitation, chemiluminescence (CL), lamp implantation, persistent luminescence nanoparticles (PLNPs), and X-rays to activate photosensitizers at the deep-seated tumor site [24]. Nanocarriers possess the capability of targeting active cancer cell or mitochondria which improves the photosensitizer accumulation at specific malignant cells even in the deep-seated tumors [25]. Moreover, recent combination of immunotherapy and the use of macrophages as an immunocyte-based drug delivery system to enhance chemotherapy, PDT, as well as immunotherapy, improved the effectiveness of the treatment of deep-seated tumors such as bone metastases [24]. Therefore, it is critical for safety and effectiveness purposes to analyze the PDT influence and effect on metastatic lesions.

In this study, we aimed to investigate the effects of PDT treatment on both primary and human metastatic cancer cell lines. Particularly, we investigated both qualitatively and quantitatively the effect of PDT exposure with 5-ALA on cellular migration/invasiveness potential, viability, apoptosis, and senescence growth arrest.

## 2. Material and Methods

### 2.1. Cell Lines

Two primary bone metastases cell lines and one primary bone tumor cell line were subjected to investigations. Cell lines originating from primary bone metastases were as follows: ”MAM”—a cell line derived from bone metastases of renal cell carcinoma, and ”MAC”- bone metastases of invasive ductal breast carcinoma. Cell lines originating from a primary bone tumor, “17-1012”—giant cell tumor of bone, were also employed. All cell lines were kindly provided by Dr. Sabine Schleicher from the University Children’s Hospital Tuebingen. The patients (MAM, MAC, and 17-1012 cells) had given their written informed consent to the scientific analysis and cell line establishment from tissue samples. Full Institutional and ethical approval was obtained before the commencement of the study (project no. 008/2014BO2). As controls, bone marrow-derived mesenchymal stem cells (MSC) were isolated as previously described [26] at the University Hospital Tuebingen after written informed consent of the patients (ethical committee approval—project no. 401/2013 BO2).

Cell lines: MAM, 17-1012 were cultured in RPMI (Invitrogen, Karlsruhe, Germany) supplemented with 10% (*v*/*v*) fetal bovine serum (FBS Invitrogen, Karlsruhe, Germany) and 2 mM L-glutamine (Invitrogen, Karlsruhe, Germany) and MAC and MSC cells in Dulbecco’s modified Eagle’s medium (DMEM) with GlutaMAX, 4.5 g/L D-glucose (Gibco, Life Technologies, Darmstadt, Germany) supplemented with 10% (*v*/*v*) FBS (Invitrogen, Karlsruhe, Germany). Cells were cultured at 37 °C in a humidified atmosphere with 5% CO_2_.

Routine microbiological analysis to assess the absence of mycoplasma contamination was carried out using a PCR Mycoplasma Detection Kit following the manufacturer’s instruction (Applied Biosystems, Darmstadt, Germany).

### 2.2. PDT Exposure with 5-Aminolaevulinic Acid

An amount of 2 × 10^4^ cells—MAC, 1 × 10^4^ cells—MAM, 3 × 10^4^—17-1012, and 2.5 × 10^4^—MSCs cells was seeded into each 2-chamber culture inserts of 6-well plates with opaque F-bottom (ibidi GmbH, Gräfelfing, Germany). After 24 h incubation at 37 °C, 5% CO_2_, the insets were removed, and the culture medium was replaced with a new one containing either 1 mM or 1.5 mM of 5-aminolaevulinic acid (5-ALA) photosensitizer. The cells were subjected to PDT exposure—blue light (436 nm, 36 J/cm^2^, IlluminOss Medical Inc., East Providence, RI, USA) in a continuous output mode for predefined time frames of 300 s and 2000 s. Cells that were not subjected to PDT exposure served as controls.

### 2.3. Migration Assay

Following PDT exposure, the cells (see section: “PDT exposure with 5-aminolaevulinic acid”) were fixed with 4% (*w*/*v*) paraformaldehyde in phosphate-buffered saline (PBS, Sigma-Aldrich, Darmstadt, Germany) for 30 min, and stained with 0.5% (*w*/*v*) Coomassie Brilliant Blue R250 (Sigma-Aldrich, Darmstadt, Germany) in 100% (*v*/*v*) methanol (VWR Fontenay-Sous-Bois, France) for 30 min. The migration of cells into gaps was visualized and photographed using an inverse phase-contrast microscope (Leica DM IMBRE, Wetzlar, Germany) and quantified using the open-source software ImageJ (version: 1.53a, https://imagej.net/Downloads, National Institutes of Health, Bethesda, MD, USA). The experiments were repeated three time for each cell type and exposure condition.

### 2.4. Viability Assessment

The cellular viability after PDT treatment was assessed using a (3-(4,5-dimethylthiazol-2-yl)-5-(3-carboxymethoxyphenyl)-2-(4-sulfophenyl)-2H-tetrazolium) MTS colorimetric assay (Promega CellTiter 96^®^ Aqueous One Solution, Madison, WI, USA). Principally, cell viability determination was based on the mitochondrial conversion of MTS to soluble formazan, indicative of the number of viable cells [27]. Following PDT exposure, seeded cells (1.5 × 10^4^ cells for each cell type) were supplemented with 50 μL of a 2 mg/mL solution of MTS in complete media and incubated for 90 min at 37 °C, 5% CO_2_. The absorbance was measured with an EL800 microplate reader (BioTek Instruments GmbH, Bad Friedrichshall, Germany) at a wavelength of 490 nm. Each experiment was conducted in triplicates and was repeated three times.

### 2.5. Nuclear Morphology Assessment

A qualitative nuclear morphology assessment was done as previously described [28]. Briefly, cells were fixed with ice-cold 100% (*v*/*v*) methanol (VWR Fontenay-Sous-Bois, France) for 10 min. Afterward, cells were washed with PBS and permeabilized with 0.2% (*v*/*v*) Triton X-100 (Carl Roth, Karlsruhe, Germany) in PBS. Nuclear staining was performed with 1% (*w*/*v*) 4′,6-diamidino-2-phenylindole (DAPI, Life Technologies, Darmstadt, Germany) in PBS for 5 min. As a green-fluorescent cytosolic stain, 500 nm of CellTracker™ Green (Thermo Scientific, Waltham, MA, USA) was added onto the cells for 30 min. Fluorescence-stained cells were visualized with a Carl Zeiss Observer Z1 fluorescence microscope (Carl Zeiss Microscopy, Jena, Germany). Staurosporine (1 µM for 6 h at 37 °C, 5% CO_2_, Abcam, Cambridge, UK) treated cells were included as a positive control (for apoptosis induction validation) [29]. Each experiment was repeated three times per cell type and exposure condition.

### 2.6. Senescence Assay

In order to investigate if PDT exposure triggers a state of stable cell cycle arrest, following PDT irradiation the seeded cells (1.5 × 10^4^ cells for each cell type) were subjected to a quantitative senescence-associated ß-galactosidase assay (Quantitative Cellular Senescence Assay #CBA-231, Biotrend Chemikalien GmbH, Köln, Germany) following the manufacturer’s instructions. The absorbance was measured with a GM3510 fluorescence microplate reader (GlowMAx^®^, Promega, Madison, WI, USA) at 360 nm (Excitation)/465 nm (Emission). Three independent measurements were performed for each cell type and exposure condition.

### 2.7. Statistical Analysis

The data are either graphically displayed as median and displayed as boxplots, or as mean with standard error of the mean (SEM) and graphically displayed as bar diagrams. As the seeding density was adjusted for the migration assay in order to observe an effect, due to different seeding densities we refrained from performing statistical analysis. For the quantitative assays, comparison between experimental results was performed by one-way analysis of variance and t-test for independent samples as a post hoc test. Alpha adjustment based on a significance level of 0.05 was performed using the Bonferroni method. Statistical analysis was performed with SPSS Statistics 22 (IBM Corp., Armonk, NY, USA). As the seeding density was adjusted for the migration assay in order to observe an effect, due to different seeding densities, we refrained from performing statistical analysis.

## 3. Results

### 3.1. Migration Assay

**Figure 1:**
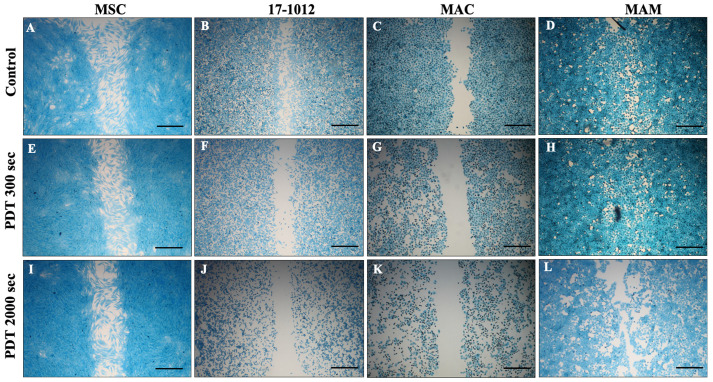
**Effect of PDT exposure on primary bone tumor and metastatic bone cell lines**. All cell lines (17-1012—giant cell tumor of bone (**B**,**F**,**J**), MAC—bone metastases of invasive ductal breast carcinoma (**C**,**G**,**K**), MAM—bone metastases of renal cell carcinoma (**D**,**H**,**L**), and MSC as a control (**A**,**E**,**I**) were subjected to PDT exposure for predefined time frames of either 300 s or 2000 s with 5-ALA as a photosensitizer at a concentration of 1.5 mM). Ten-fold magnification, scale bar (black) represents 300 µm. Abbreviations: 5-ALA—5-aminolaevulinic acid, PDT—photodynamic therapy, sec—seconds.

**Figure 2:**
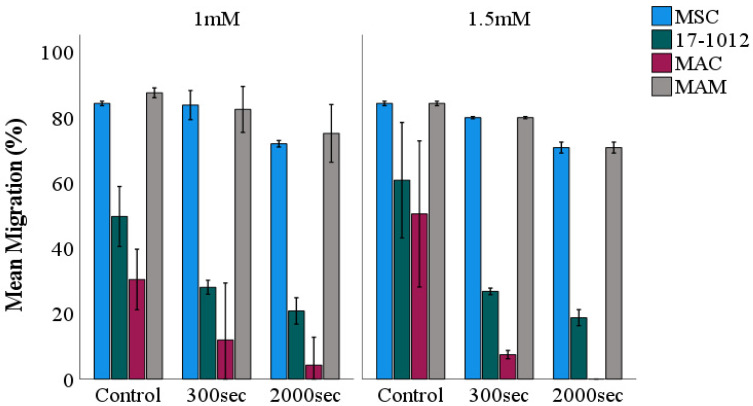
**Semi-quantitative assessment of cellular migration potential following PDT exposure**. The rate of cell migration for all cell lines (17-1012—giant cell tumor of bone, MAC—bone metastases of invasive ductal breast carcinoma, MAM—bone metastases of renal cell carcinoma and MSC) was measured as a distance (μm, using the open-source software ImageJ) 24 h post light exposure. As a photosensitizer, 5-ALA was employed at two different concentrations: 1 mM (left side) and 1.5 mM (right side). Values represent means ± SEM; *n* = 3 independent experiments. Abbreviations: 5-ALA—5-aminolaevulinic acid, sec—seconds.

The MSC-control group showed no effect after 5-ALA PDT irradiation (Figure 1A,E,I; Suppl. Figure S1A,B). In contrast, the MAC (Figure 1C,G,K; Figure 2) and 17-1012 (Figure 1B,F,J; Figure 2) cells showed a decreasing tendency with respect to migration potential after sensitizing with 5-ALA (1.5 mM) and 300 s, respectively, and 2000 s light exposure when compared with the non-irradiated breast cancer cells. This effect was not observed in bone metastases of renal carcinoma (MAM), which did not show notable changes neither in cell migration nor cell morphology after 5-ALA and additional irradiation with blue light (Figure 1D,H,L; Suppl. Figure S1G,H). A minor decrease in cell migration was observed after longer (2000 s) PDT exposure (Figure 1L; Figure 2), and a similar tendency was observed for the PDT sensitizing with 5-ALA at a concentration of 1 mM (Suppl. Figure S2).

A decrease in cell density, reduction in cytoplasm, and a smaller, altered morphology were observed for 17-1012 and MAC cells (Figure 1F,J,G,K) when compared with their corresponding controls (Figure 1B,C; Suppl. Figure S1C,E), and this effect was more pronounced with higher exposure time (Figure 1; Suppl. Figure S1D,F).

### 3.2. Viability Assessment

**Figure 3:**
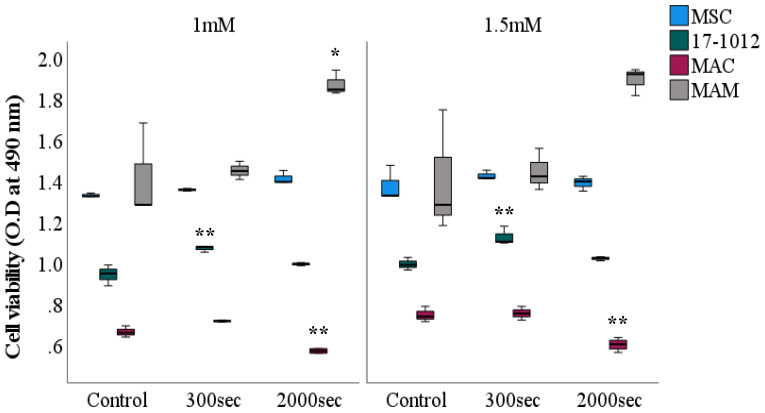
**MTS—viability assessment of bone metastases cell lines after PDT exposure**. All cell lines (17-1012—giant cell tumor of bone, MAC—bone metastases of invasive ductal breast carcinoma, MAM—bone metastases of renal cell carcinoma, and MSC as a control) were subjected to PDT exposure for predefined time frames of either 300 s or 2000 s. As a photosensitizer, 5-ALA was employed at two different concentrations: 1 mM (left side) and 1.5 mM (right side). Comparisons were conducted in relation to the control group (cells that were not subjected to PDT exposure). Data of 3 experiments performed in triplicates. * *p* < 0.05, ** *p* < 0.01. Abbreviations: 5-ALA—5-aminolaevulinic acid, O. D—optical density, sec—seconds.

To investigate the effect on cellular viability after PDT exposure with 5-ALA, an MTS assay was conducted on all cell lines. Independent of the exposure time, for both of the employed 5-ALA concentrations (1 mM and 1.5 mM) a similar and comparable tendency for all of the cell lines was observed (Figure 3). A statistically significant decrease in viability was observed for the MAC cell line at both 5-ALA concentrations: 1 mM (*p* = 0.009) and 1.5 mM (*p* = 0.002) at a 2000 s exposure time when compared with their corresponding controls. Contrastingly, the 17-1012 cell line exhibited an increase in viability (*p* = 0.007, both 5-ALA concentrations) at an exposure of 300 s, followed by a decrease at 2000 s of exposure time (*p* = 0.257). The MAM cell line showed an increasing tendency in viability for both exposure times and 5-ALA concentrations (*p* = 0.022, 1 mM, 2000 s, respectively, *p* = 0.056, 1.5 mM, 2000 s). Irrespective of the exposure time and photosensitizer concentration, the number of viable MSCs remained unaltered and no notable change was observed.

### 3.3. Fluorescence Assessment of Apoptosis

**Figure 4:**
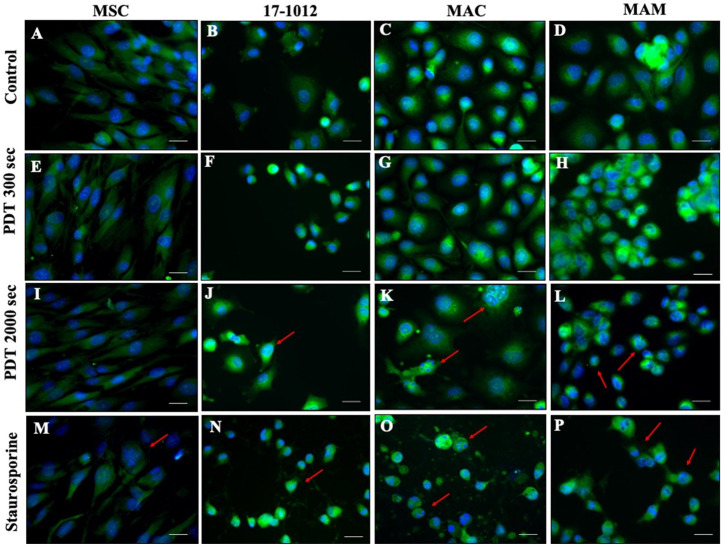
**Representative figures of nuclear fragmentation assessment after PDT exposure with 5-ALA as a photosensitizer at a concentration of 1.5 mM**. All cell lines (MSC—bone marrow-derived mesenchymal stem cells (**A**,**E**,**I**), 17-1012—giant cell tumor of bone (**B**,**F**,**J**), MAC—bone metastases of invasive ductal breast carcinoma (**C**,**G**,**K**), MAM—bone metastases of renal cell carcinoma (**D**,**H**,**L**) were subjected to PDT exposure for predefined time frames of either 300 s or 2000 s). Staurosporine positive controls were included for cell lines (MSC: M, 17-1012: N, MAC: O, MAM: P). 40-fold magnification, scale bar (white) represents 50 µm. Red arrows pinpoint nuclear fragmentation. Pictures are representative of three independent experiments. Cells not subjected to PDT served as a control. Abbreviations: 5-ALA—5-aminolaevulinic acid, PDT—photodynamic therapy.

Changes in nuclear morphology as a hallmark for apoptosis were also examined qualitatively utilizing fluorescence microscopy. DAPI and cytosolic staining were used to visualize the changes in the nucleus and formation of apoptotic bodies—important hallmarks of apoptosis. It could be noted that after 2000 s of PDT exposure with 1.5 mM of 5-ALA, MAC, MAM, and 17-1012 cell lines (Figure 4 and Supp. Figure S3 for 1 mM 5-ALA) showed apoptotic features at various extents, such as nuclear shrinkage (red arrows, Figure 4). Nuclear fragmentation was particularly notable in the MAC cell line (Figure 4K). It has to be noted that this distinct morphological feature—characteristic for apoptosis—was not a generalized feature for the entire cell population. The MSC-control group showed no effect after 5-ALA PDT irradiation (Figure 4A,E,I,M).

### 3.4. Cellular Senescence

**Figure 5:**
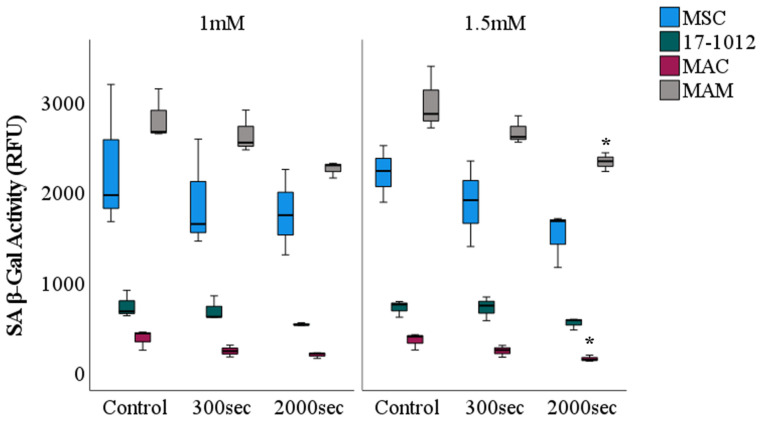
**Cellular senescence (SA β-Gal Activity**) **assessment of bone metastasis cell lines after PDT exposure**. All cell lines (17-1012—giant cell tumor of bone, MAC—bone metastases of invasive ductal breast carcinoma, MAM—bone metastases of renal cell carcinoma, and MSC as a control) were subjected to PDT exposure for predefined time frames of either 300 s or 2000 s. As a photosensitizer, 5-ALA was employed at two different concentrations: 1 mM (left side) and 1.5 mM (right side). Comparisons were conducted in relation to the control group (cells that were not subjected to PDT exposure). Data of 3 experiments performed in triplicates. * *p* < 0.05. Abbreviations: 5-ALA—5-aminolaevulinic acid, RFU—relative fluorescence units, sec—seconds.

Cellular senescence or cellular aging is characterized by a diminished replicative capacity coupled with altered functionality within the cell [30]. To clarify and elucidate the cause of the observed changes in cellular vitality and nuclear fragmentation after PDT exposure, all cell lines before and post PDT were sequentially evaluated in terms of cellular arrest (SA β-Gal activity). For both of the employed 5-ALA concentrations (1 mM and 1.5 mM) a similar and comparable decreasing tendency in SA β-Gal activity was noted for all of the cell lines (Figure 5). More so, all cell lines were characterized by a decrease of SA β-Gal activity with increasing exposure time. Overall, a higher fluorescence intensity signal representative for β-Gal activity was noted for the MAM cell line as well as the control group, MSC, when compared with the MAC and 17-1012 cell lines.

## 4. Discussion

Currently, radiotherapy is the gold standard in the treatment of metastases; however, it only offers a limited capacity for tissue repair and is related to severe adverse effects. Alternate approaches for treating tumors and metastases are urgently needed and one such potential candidate is the combination of photosensitizer, 5-aminolevulinic acid (5-ALA), and photodynamic therapy (PDT); however, the data on its safety and effectiveness in the treatment of metastatic lesions is limited. 5-ALA PDT offers several advantages, such as low toxicity, fast clearance, and rapid conversion to porphyrin.

In this study, we assessed 5-ALA PDT treatment against different human cell lines, primary giant cell tumor of bone and bone metastases of invasive ductal carcinoma, renal carcinomas, as well as MSC (control group). We found that different human cell lines have distinct 5-ALA-PDT-sensitivities. Exposure of cells to 5-ALA PDT proved particularly effective in decreasing the migration potential, viability, cell proliferation, and density in bone metastases of invasive ductal carcinoma. For cells derived from giant cell tumor of bone, 5-ALA PDT irradiation triggered a notable decrease in migration potential, and cell density. No notable effect was observed in bone metastases of renal carcinoma and MSC group. The reduction effect, was, in general, more pronounced with a longer PDT exposure time and higher 5-ALA concentration suggesting a dose and exposure-dependent effect consistent with the previous research [31,32]. Numerous studies showed that at the cellular level, 5-ALA PDT induces multiple cell death subroutines [32,33,34,35], resulting in downstream activating both apoptotic and necrotic pathways. Additionally, while invasive ductal carcinoma and giant cell tumor presented overall lower cell viability compared with the other two cell lines, the giant cell tumor firstly showed increased viability after shorter (300 s) PDT exposure. Even after a long PDT exposure, no significant effect on cell viability was observed. In turn, for the bone metastases of invasive ductal carcinoma, 5-ALA PDT proved effective in synergistically inhibiting the cell viability. This interesting observation could be attributed to the lower 5-ALA PDT sensitivity of giant cell tumor cell line—insufficient to induce the significant apoptotic effect. No notable effect in cell viability was observed for the rest of the cell lines.

In fact, the cell line with lower sensitivity to 5-ALA PDT, that is, bone metastases of renal carcinoma (MAM), actually went into the state of cellular senescence caused by 5-ALA PDT exposure, and no therapeutic effect was observed. Senescence-induced cell lines showed no significant changes in cell migration even after prolonged PDT irradiation, and in fact exhibited increased viability even after a long exposure time. Thus, different sensitivity to the same doses and exposure of 5-ALA and PDT results in a completely different cell fate. While cellular senescence may be a beneficial strategy preventing the growth of the tumor short-term, cells remain metabolically active, affecting neighboring cells and stimulating cancer-promoting mechanisms [36,37,38,39,40]. At the same time, the lack of PDT-induced apoptosis in the healthy MSC cell line confirms the safety and selectivity of the treatment. In addition, previous studies showed that PDT treatment also improved differentiation capabilities at a low exposure dose [41]. As such, we confirm that 5-ALA PDT has potential applicability in primary giant cell tumor of bone and bone metastases of invasive ductal carcinoma. All cancer cell lines showed some hallmarks of apoptosis and nuclear shrinkage while nuclear fragmentation was particularly pronounced in bone metastases of invasive ductal carcinoma cell line consistent with the highest sensitivity to 5-ALA PDT. Even though all cell lines had apoptotic features at 2000 s, it was not a generalized feature—in the 17-1012 cell line it was just a minor effect.

In bone metastases of the renal carcinoma cell line, 5-ALA PDT-induced senescence could be attributed to a too-low light dose, as the cellular decision between apoptosis/necrosis and senescence partially depends on it [42,43]. Our results suggest that different cell lines may have a different sensitivity to the same doses and exposure of 5-ALA PDT results in apoptosis or senescence of two different cell fates, which is consistent with previous research [44].

Even though nuclear morphologic changes such as chromosome condensation and fragmentation are considered hallmarks of apoptosis [45], DNA fragmentation alone is not a mandatory event for apoptotic cell death or representative of the entire cell population [46]. Additionally, PDT might actually induce a transient inhibitory effect [47] as the cells were eventually returning to their basal viability levels. In addition, as PDT emerges as a first-line therapy, it is important to also consider the resistance mechanism. While DNA-induced damage may induce senescence and temporary decrease in migration potential, the very same process may help the remaining cancer cells to escape therapy. Nevertheless, this resistance mechanism is extremely limited in PDT-treated cells [48]. Further in-depth analysis of PDT-induced apoptotic/senescence effects is called for.

One of the key advantages of PDT is that it can be used either before or after chemotherapy, radiotherapy, or surgery, without compromising these therapeutic modalities and the adverse effects of chemotherapy or radiation do not affect sensitivity to PDT. Several in vitro studies have shown the synergistic benefit of PDT and ionizing radiation in several pathologies [49,50,51,52]. Therefore, further research is warranted to assess combination therapy with radiotherapy, chemotherapy, and to optimize the protocol for maximal disease clearance as well as to elucidate the actual PDT therapeutic potential in other cancer pathologies in in vivo animal studies.

## 5. Conclusions

Human cell lines have different sensitivity to the same doses and exposure of 5-ALA PDT, resulting in two different cell fates apoptosis or senescence, depending on the extent of the cellular damage. We found that PDT has potential applicability in bone metastases of invasive ductal carcinoma. While no possible therapeutic effect was observed in bone metastases of renal carcinoma, a limited effect on primary bone tumors (giant cell bone tumor) is conceivable.

## 6. Study Limitations

While our study offers an interesting new perspective to the in vitro effects of 5-ALA PDT irradiation on metastatic cell lines, the approach implies a laborious work. For these reasons, only three independent experiments for each cell line were finally conducted. This aspect should be kept in mind when interpreting the statistical results, however, the measured tendency should not be affected. Additionally, it has to be noted that cellular heterogeneity in tumor cells is a well-established phenomenon [53,54] considered to be an important cause of drug resistance that hinders the treatment outcome [55,56]. Even though in our study we analyzed only one patient-derived cell line for each neoplasia type, this might not representative the entire neoplastic cell population. Our findings are, however, highly consistent with the previous publications that analyzed and showed a differential sensitivity to PDT irradiation in various cancer-derived lines [44,57,58].

## Data Availability

All data can be obtained from authors on a reasonable request.

## Supplementary Materials

The following are available online at https://www.mdpi.com/article/10.3390/biology10101020/s1. Figure S1. Effect of PDT exposure on the morphology of primary bone tumor and metastatic bone cell lines. Figure S2. Effect of PDT exposure with on primary bone tumor and metastatic bone cell lines.

## References

[1] Coleman R.E. (2001). Metastatic bone disease: Clinical features, pathophysiology and treatment strategies. Cancer Treat. Rev..

[2] Janjan N., Lutz S.T., Bedwinek J.M., Hartsell W.F., Ng A., Pieters R.S., Ratanatharathorn V., Silberstein E.B., Taub R.J., Yasko A.W. (2009). Therapeutic guidelines for the treatment of bone metastasis: A report from the American College of Radiology Appropriateness Criteria Expert Panel on Radiation Oncology. J. Palliat. Med..

[3] Milano M.T., Constine L.S., Okunieff P. (2007). Normal tissue tolerance dose metrics for radiation therapy of major organs. Semin. Radiat. Oncol..

[4] Schmeler K.M., Jhingran A., Iyer R.B., Sun C.C., Eifel P.J., Soliman P.T., Ramirez P.T., Frumovitz M., Bodurka D.C., Sood A.K. (2010). Pelvic fractures after radiotherapy for cervical cancer: Implications for survivors. Cancer.

[5] Ahn T.G., Lee B.R., Choi E.Y., Kim D.W., Han S.J. (2012). Photodynamic therapy for breast cancer in a BALB/c mouse model. J. Gynecol. Oncol..

[6] Rizvi I., Celli J.P., Evans C.L., Abu-Yousif A.O., Muzikansky A., Pogue B.W., Finkelstein D., Hasan T. (2010). Synergistic Enhancement of Carboplatin Efficacy with Photodynamic Therapy in a Three-Dimensional Model for Micrometastatic Ovarian Cancer. Cancer Res..

[7] Agostinis P., Berg K., Cengel K.A., Foster T.H., Girotti A.W., Gollnick S.O., Hahn S.M., Hamblin M.R., Juzeniene A., Kessel D. (2011). Photodynamic therapy of cancer: An update. CA Cancer J. Clin..

[8] Dolmans D.E., Fukumura D., Jain R.K. (2003). Photodynamic therapy for cancer. Nat. Rev. Cancer.

[9] Dougherty T.J. (1987). Photosensitizers: Therapy and Detection of Malignant Tumors. Photochem. Photobiol..

[10] Burch S., Bogaards A., Siewerdsen J., Moseley D., Yee A., Finkelstein J., Weersink R., Wilson B., Bisland S. (2005). Photodynamic therapy for the treatment of metastatic lesions in bone: Studies in rat and porcine models. J. Biomed. Opt..

[11] Stewart F., Baas P., Star W. (1998). What does photodynamic therapy have to offer radiation oncologists (or their cancer patients)?. Radiother. Oncol. J. Eur. Soc. Ther. Radiol. Oncol..

[12] Malik Z., Lugaci H. (1987). Destruction of erythroleukaemic cells by photoactivation of endogenous porphyrins. Br. J. Cancer.

[13] Abrahamse H., Hamblin M.R. (2016). New photosensitizers for photodynamic therapy. Biochem. J..

[14] Dalton J.T., Yates C.R., Yin D., Straughn A., Marcus S.L., Golub A.L., Meyer M.C. (2002). Clinical pharmacokinetics of 5-aminolevulinic acid in healthy volunteers and patients at high risk for recurrent bladder cancer. J. Pharmacol. Exp. Ther..

[15] Shetty T., Corson T.W. (2020). Mitochondrial Heme Synthesis Enzymes as Therapeutic Targets in Vascular Diseases. Front. Pharmacol..

[16] Schwartz C., Rühm A., Tonn J.-C., Kreth S., Kreth F.-W. (2015). SURG-25: Interstitial Photodynamic Therapy Of De-Novo Glioblastoma Multiforme WHO IV. Neuro Oncol..

[17] Vermandel M., Dupont C., Quidet M., Lecomte F., Lerhun E., Mordon S., Betrouni N., Reyns N. (2017). Set-up of the first pilot study on intraopertive 5-ALA PDT: INDYGO trial. Photodiagnosis Photodyn. Ther..

[18] Wiedmann M., Caca K., Berr F., Schiefke I., Tannapfel A., Wittekind C., Mössner J., Hauss J., Witzigmann H. (2003). Neoadjuvant photodynamic therapy as a new approach to treating hilar cholangiocarcinoma: A phase II pilot study. Cancer.

[19] Sultan S.M., El-Doray A.A., Hofstetter A., Abdel-Gawad O., El-Mahdy Ael D., Khoder W. (2006). Photodynamic selectivity of 5-aminolevulinic acid to prostate cancer cells. J. Egypt. Natl. Cancer Inst..

[20] van Straten D., Mashayekhi V., de Bruijn H.S., Oliveira S., Robinson D.J. (2017). Oncologic Photodynamic Therapy: Basic Principles, Current Clinical Status and Future Directions. Cancers.

[21] Fan H.T., Wang L., Zhang P., Liu S.B. (2015). Photodynamic therapy in spinal metastases: A qualitative analysis of published results. Int. Surg..

[22] Wise-Milestone L., Akens M.K., Lo V.C., Yee A.J., Wilson B.C., Whyne C.M. (2012). Local treatment of mixed osteolytic/osteoblastic spinal metastases: Is photodynamic therapy effective?. Breast Cancer Res. Treat..

[23] Fisher C., Ali Z., Detsky J., Sahgal A., David E., Kunz M., Akens M., Chow E., Whyne C., Burch S. (2019). Photodynamic Therapy for the Treatment of Vertebral Metastases: A Phase I Clinical Trial. Clin. Cancer Res..

[24] Li W.-P., Yen C.-J., Wu B.-S., Wong T.-W. (2021). Recent Advances in Photodynamic Therapy for Deep-Seated Tumors with the Aid of Nanomedicine. Biomedicines.

[25] Liu Y., Meng X., Wang H., Tang Z., Zuo C., He M., Bu W. (2018). Photoelectron Transfer at ZnTPyP Self-Assembly/TiO(2) Interfaces for Enhanced Two-Photon Photodynamic Therapy. ACS Appl. Mater. Interfaces.

[26] Battula V.L., Treml S., Bareiss P.M., Gieseke F., Roelofs H., de Zwart P., Müller I., Schewe B., Skutella T., Fibbe W.E. (2009). Isolation of functionally distinct mesenchymal stem cell subsets using antibodies against CD56, CD271, and mesenchymal stem cell antigen-1. Haematologica.

[27] Riss T.L., Moravec R.A., Niles A.L., Duellman S., Benink H.A., Worzella T.J., Minor L., Markossian S., Grossman A., Brimacombe K., Arkin M., Auld D., Austin C.P., Baell J., Chung T.D.Y., Coussens N.P., Dahlin J.L. (2004). Cell Viability Assays. Assay Guidance Manual.

[28] Mandelkow R., Gümbel D., Ahrend H., Kaul A., Zimmermann U., Burchardt M., Stope M.B. (2017). Detection and Quantification of Nuclear Morphology Changes in Apoptotic Cells by Fluorescence Microscopy and Subsequent Analysis of Visualized Fluorescent Signals. Anticancer. Res..

[29] Belmokhtar C.A., Hillion J., Ségal-Bendirdjian E. (2001). Staurosporine induces apoptosis through both caspase-dependent and caspase-independent mechanisms. Oncogene.

[30] Beauséjour C. (2007). Bone marrow-derived cells: The influence of aging and cellular senescence. Handb. Exp. Pharmacol..

[31] Chen X., Zhao P., Chen F., Li L., Luo R. (2011). Effect and mechanism of 5-aminolevulinic acid-mediated photodynamic therapy in esophageal cancer. Lasers Med. Sci..

[32] Bacellar I.O., Tsubone T.M., Pavani C., Baptista M.S. (2015). Photodynamic Efficiency: From Molecular Photochemistry to Cell Death. Int. J. Mol. Sci..

[33] dos Santos A.F., de Almeida D.R.Q., Terra L.F., Baptista M.S., Labriola L. (2019). Photodynamic therapy in cancer treatment—An update review. J. Cancer Metastasis Treat..

[34] Amo T., Kawanishi N., Uchida M., Fujita H., Oyanagi E., Utsumi T., Ogino T., Inoue K., Shuin T., Utsumi K. (2009). Mechanism of cell death by 5-aminolevulinic acid-based photodynamic action and its enhancement by ferrochelatase inhibitors in human histiocytic lymphoma cell line U937. Cell Biochem. Funct..

[35] Chelakkot V.S., Som J., Yoshioka E., Rice C.P., Rutihinda S.G., Hirasawa K. (2019). Systemic MEK inhibition enhances the efficacy of 5-aminolevulinic acid-photodynamic therapy. Br. J. Cancer.

[36] Gordon R.R., Nelson P.S. (2012). Cellular senescence and cancer chemotherapy resistance. Drug Resist. Updates Rev. Comment Antimicrob. Anticancer. Chemother..

[37] Shay J.W., Roninson I.B. (2004). Hallmarks of senescence in carcinogenesis and cancer therapy. Oncogene.

[38] Lee M., Lee J.S. (2014). Exploiting tumor cell senescence in anticancer therapy. BMB Rep..

[39] Mosieniak G., Strzeszewska A. (2014). The role of cellular senescence in carcinogenesis and antitumor therapy. Postepy Biochem..

[40] Coppé J.P., Desprez P.Y., Krtolica A., Campisi J. (2010). The senescence-associated secretory phenotype: The dark side of tumor suppression. Annu. Rev. Pathol..

[41] Fekrazad R., Asefi S., Khorsandi K., Nejatifard M. (2020). Photo biostimulatory effect of low dose photodynamic therapy on human mesenchymal stem cells. Photodiagnosis Photodyn. Ther..

[42] Grigalavicius M., Juraleviciute M., Kwitniewski M., Juzeniene A. (2017). The influence of photodynamic therapy with 5-aminolevulinic acid on senescent skin cancer cells. Photodiagnosis Photodyn. Ther..

[43] Castano A.P., Demidova T.N., Hamblin M.R. (2005). Mechanisms in photodynamic therapy: Part two-cellular signaling, cell metabolism and modes of cell death. Photodiagnosis Photodyn. Ther..

[44] Perry R.R., Matthews W., Mitchell J.B., Russo A., Evans S., Pass H.I. (1990). Sensitivity of different human lung cancer histologies to photodynamic therapy. Cancer Res..

[45] Wickman G., Julian L., Olson M.F. (2012). How apoptotic cells aid in the removal of their own cold dead bodies. Cell Death Differ..

[46] Schulze-Osthoff K., Walczak H., Dröge W., Krammer P.H. (1994). Cell nucleus and DNA fragmentation are not required for apoptosis. J. Cell Biol..

[47] Tong Z., Singh G., Rainbow A.J. (2002). Sustained activation of the extracellular signal-regulated kinase pathway protects cells from photofrin-mediated photodynamic therapy. Cancer Res..

[48] Casas A., Di Venosa G., Hasan T., Al B. (2011). Mechanisms of resistance to photodynamic therapy. Curr. Med. Chem..

[49] Kim Y.W., Bae S.M., Battogtokh G., Bang H.J., Ahn W.S. (2012). Synergistic anti-tumor effects of combination of photodynamic therapy and arsenic compound in cervical cancer cells: In vivo and in vitro studies. PLoS ONE.

[50] Morton C.A., Szeimies R.-M., Basset-Séguin N., Calzavara-Pinton P.G., Gilaberte Y., Hædersdal M., Hofbauer G.F.L., Hunger R.E., Karrer S., Piaserico S. (2020). European Dermatology Forum guidelines on topical photodynamic therapy 2019 Part 2: Emerging indications—Field cancerization, photorejuvenation and inflammatory/infective dermatoses. J. Eur. Acad. Dermatol. Venereol..

[51] Hamblin M.R., Hasan T. (2004). Photodynamic therapy: A new antimicrobial approach to infectious disease?. Photochem. Photobiol. Sci..

[52] Wang X., Hu J., Wang P., Zhang S., Liu Y., Xiong W., Liu Q. (2015). Analysis of the in vivo and in vitro effects of photodynamic therapy on breast cancer by using a sensitizer, sinoporphyrin sodium. Theranostics.

[53] Huxley J. (1958). Biological aspects of cancer: Harcourt, Brace. Science.

[54] Alizadeh A.A., Aranda V., Bardelli A., Blanpain C., Bock C., Borowski C., Caldas C., Califano A., Doherty M., Elsner M. (2015). Toward understanding and exploiting tumor heterogeneity. Nat. Med..

[55] McGranahan N., Swanton C. (2017). Clonal Heterogeneity and Tumor Evolution: Past, Present, and the Future. Cell.

[56] Dai Z., Gu X.-y., Xiang S.-y., Gong D.-d., Man C.-f., Fan Y. (2020). Research and application of single-cell sequencing in tumor heterogeneity and drug resistance of circulating tumor cells. Biomark. Res..

[57] Barron G.A., Moseley H., Woods J.A. (2013). Differential sensitivity in cell lines to photodynamic therapy in combination with ABCG2 inhibition. J. Photochem. Photobiol. B Biol..

[58] Choi B.-h., Ryoo I.-g., Kang H.C., Kwak M.-K. (2014). The Sensitivity of Cancer Cells to Pheophorbide a-Based Photodynamic Therapy Is Enhanced by NRF2 Silencing. PLoS ONE.

