# Supplementary material for: Assessment of 5-Aminolevulinic Acid-Mediated Photodynamic Therapy on Bone Metastases: An in Vitro Study
Source: Biology (Basel). 2021 Oct 9;10(10):1020. doi: 10.3390/biology10101020 (PMC8533584; doi:10.3390/biology10101020)
Supplement: Supplementary file 1 [file biology-10-01020-s001.zip › biology-1395547-supplementary.pdf]

Online supplement

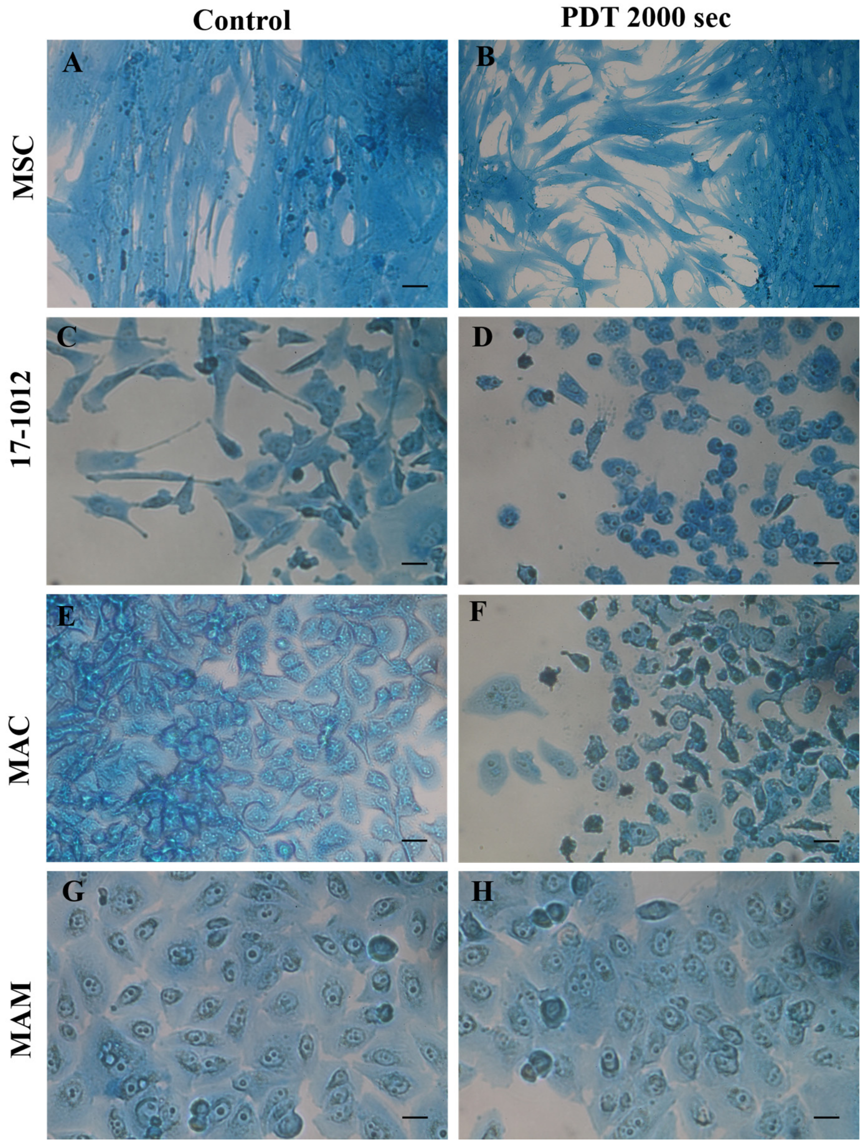

Figure S1. Effect of PDT exposure on the morphology of primary bone tumor and metastatic bone cell lines. All cell lines (17-1012 - giant cell tumor of bone (C, D), MAC - bone metastases of invasive ductal breast carcinoma (E, F), MAM - bone metastases of renal cell carcinoma (G, H) and MSC as a control (A, B)) were subjected to PDT exposure for a predefined time frame of 2000 sec with 5-ALA as a photosensitizer at a concentration of 1.5 mM. Following 5-ALA PDT exposure, the cellular migration potential and morphology were analyzed. 20-fold magnification, scale bar (black) represents 50 µm. Abbreviations: 5-ALA - 5-aminolaevulinic acid, PDT - photodynamic therapy, sec - seconds.

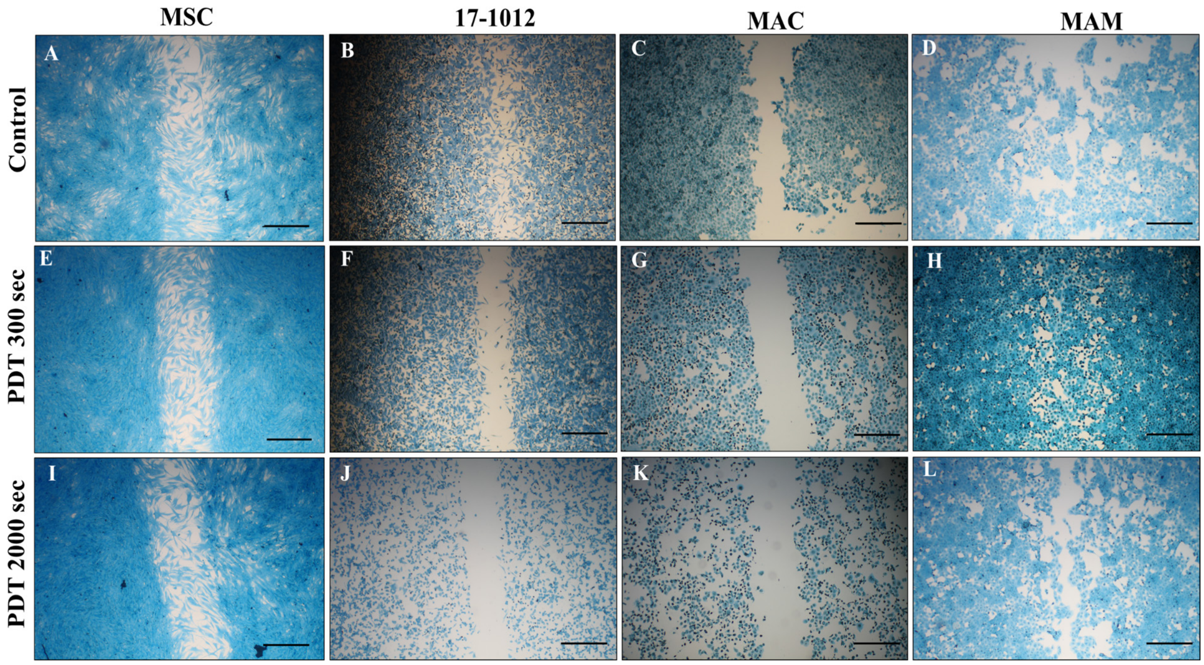

Figure S2. Effect of PDT exposure with on primary bone tumor and metastatic bone cell lines. All cell lines (17-1012 - giant cell tumor of bone (B, F, J), MAC - bone metastases of invasive ductal breast carcinoma (C, G, K), MAM - bone metastases of renal cell carcinoma (D, H, L) and MSC as a control (A, E, I)) were subjected to PDT exposure for predefined time frames of either 300 sec or 2000 sec with 5-ALA as a photosensitizer at a concentration of 1 mM). 10-fold magnification, scale bar (black) represents 300 µm. Abbreviations: 5-ALA - 5-aminolaevulinic acid, PDT - photodynamic therapy, sec - seconds.

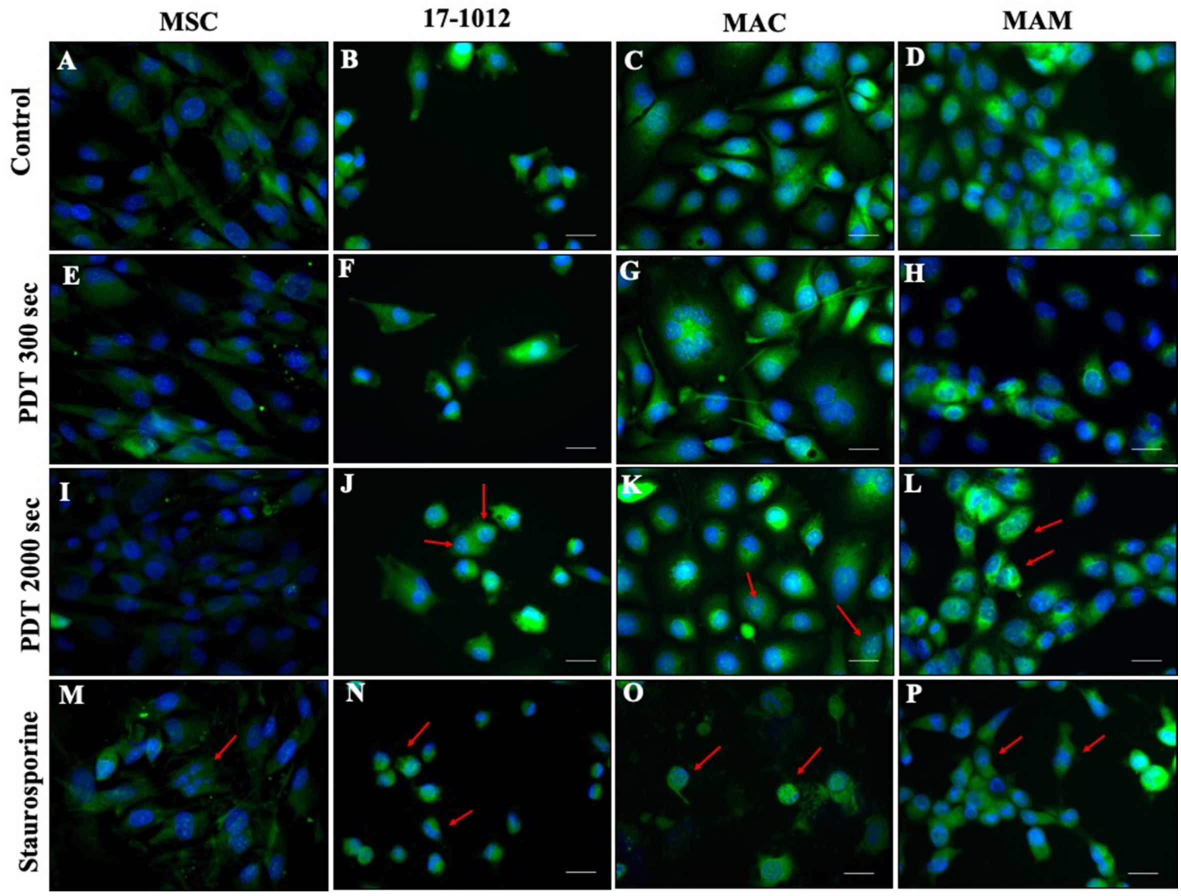

Figure S3. Representative figures of nuclear fragmentation assessment after PDT exposure with 1 mM of 5-ALA. All cell lines (MSC - bone marrow-derived mesenchymal stem cells (A, E, I), 17-1012 - giant cell tumor of bone (B, F, J), MAC - bone metastases of invasive ductal breast carcinoma (C, G, K), MAM - bone metastases of renal cell carcinoma (D, H, L)) were subjected to PDT exposure for predefined time frames of either 300 sec or 2000 sec. Cells which were not subjected to PDT exposure served as control. Red arrows indicative for nuclear fragmentation. Staurosporine positive controls were included for cell lines (MSC: M, 17-1012: N, MAC: O, MAM: P). 40-fold magnification, scale bar (white) represents 50 µm. Representative data of three independent experiments. Abbreviations: 5-ALA - 5-aminolaevulinic acid, PDT - photodynamic therapy.
